# Tumeur rénale ou pyélonéphrite xanthogranulomateuse pseudotumorale

**DOI:** 10.11604/pamj.2018.29.67.14611

**Published:** 2018-01-24

**Authors:** Siham Alaoui Rachidi, Asmae Zeriouel

**Affiliations:** 1 Service de Radiologie, Centre Hospitalier Provincial, Taounate, Maroc

**Keywords:** Rein, pyélonéphrite, xanthogranulomateuse, Kidney, pyelonephritis, xanthogranulomatous

## Abstract

We here report the case of a 55 year-old man presenting with right renal colic and with a history of intermittent lower back disorders. At the time of admission, clinical examination showed lower back pain on contact. Renal ultrasound objectified heterogeneous mass at the lower pole of the right kidney with dilation of lower calyx groups. Abdominal CT scan confirmed the presence of right inferior renal polar lesion characterized by mixed density, associated with significant hydronephrosis eroding the renal parenchyma upstream of a voluminous pyelic coralliform lithiasis. The patient underwent nephrectomy with anatomo-pathological examination revealing chronic inflammation of the renal parenchyma characterized by macrophages associated with monocytes and lymphocytes with spongy cells and fibrosis. Anatomo-pathological result was compatible with a diagnosis of xanthogranulomatous pyelonephritis. Xanthogranulomatous pyelonephritis is a relatively rare chronic pyelonephritis whose symptoms resemble those of a pseudotumor. The diagnosis is suspected on the basis of clinical arguments and on laboratory tests and may be evoked quite exclusively on the basis of preoperative CT scan. Kidney cancer was its main differential diagnosis.

## Image en médecine

Un homme âgé de 55 ans qui consulte pour une colique néphrétique droite, avec des antécédents de lombalgies intermittentes. À l'admission, on a trouvé un contact lombaire positif. L'échographie rénale a objectivé une masse hétérogène au pôle inferieur du rein droit avec une dilatation des groupes caliciels inferieurs. La tomodensitométrie abdominale a confirmé la présence d'une lésion rénale droite polaire inferieure hétérodense associée à une importante hydronéphrose laminant le parenchyme rénal en amont d'une volumineuse lithiase pyélique coralliforme. Le patient a bénéficié d'une néphrectomie dont l'analyse anatomo-pathologique a révélé un envahissement du parenchyme rénal par un infiltrat inflammatoire chronique formé de macrophages associés à des monocytes, lymphocytes avec des cellules spongieuses et de la fibrose. Cet aspect était compatible avec le diagnostic de pyélonéphrite xanthogranulomateuse. La pyélonéphrite xanthogranulomateuse est une entité de pyélonéphrite chronique relativement rare dont la symptomatologie prend souvent une forme pseudotumorale. Le diagnostic est suspecté sur un faisceau d'arguments cliniques et biologiques et peut être évoqué presque exclusivement sur l'examen tomodensitométrique préopératoire. Le cancer du rein étant son principal diagnostic différentiel.

**Figure 1:**
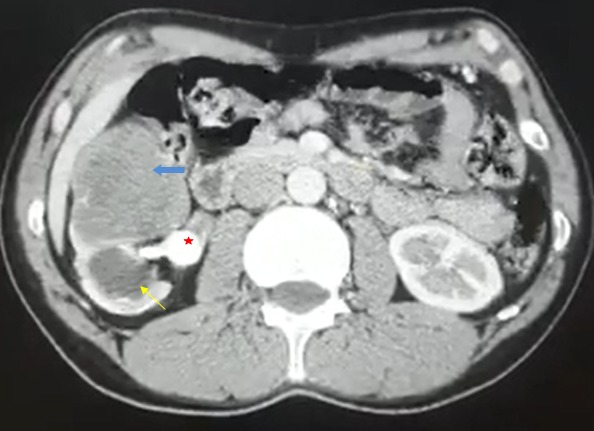
TDM abdominale en coupe axiale montrant une masse rénale droite hétérodense avec une hydronéphrose d’origine lithiasique laminant le parenchyme rénal qui est aminci. La lithiase étant pyélique coralliforme

